# Expression and localization of sterile alpha motif domain containing 5 is associated with cell type and malignancy of biliary tree

**DOI:** 10.1371/journal.pone.0175355

**Published:** 2017-04-07

**Authors:** Tomoki Yagai, Satoshi Matsui, Kenichi Harada, Fuyuki F. Inagaki, Eiko Saijou, Yasushi Miura, Yasuni Nakanuma, Atsushi Miyajima, Minoru Tanaka

**Affiliations:** 1 Laboratory of Cell Growth and Differentiation, Institute of Molecular and Cellular Biosciences, The University of Tokyo, Tokyo, Japan; 2 Department of Regenerative Medicine, Research Institute, National Center for Global Health and Medicine, Tokyo, Japan; 3 Department of Human Pathology, Kanazawa University Graduate School of Medicine, Kanazawa, Japan; 4 Department of Life Science and Medical Bio-Science, School of Advanced Science and Engineering, Waseda University, Tokyo, Japan; 5 Laboratory of Stem Cell Regulation, Institute of Molecular and Cellular Biosciences, The University of Tokyo, Tokyo, Japan; University of Navarra School of Medicine and Center for Applied Medical Research (CIMA), SPAIN

## Abstract

Cholangiocarcinoma (CC) is a type of relatively rare neoplasm in adenocarcinoma. The characteristics of CCs as well as biliary epithelial cells are heterogeneous at the different portion of the biliary tree. There are two candidate stem/progenitor cells of the biliary tree, i.e., biliary tree stem/progenitor cell (BTSC) at the peribiliary gland (PBG) of large bile ducts and liver stem/progenitor cell (LPC) at the canals of Hering of peripheral small bile duct. Although previous reports suggest that intrahepatic CC (ICC) can arise from such stem/progenitor cells, the characteristic difference between BTSC and LPC in pathological process needs further investigation, and the etiology of CC remains poorly understood. Here we show that Sterile alpha motif domain containing 5 (SAMD5) is exclusively expressed in PBGs of large bile ducts in normal mice. Using a mouse model of cholestatic liver disease, we demonstrated that SAMD5 expression was upregulated in the large bile duct at the hepatic hilum, the extrahepatic bile duct and PBGs, but not in proliferating intrahepatic ductules, suggesting that SAMD5 is expressed in BTSC but not LPC. Intriguingly, human ICCs and extrahepatic CCs exhibited striking nuclear localization of SAMD5 while the normal hilar large bile duct displayed slight-to-moderate expression in cytoplasm. *In vitro* experiments using siRNA for *SAMD5* revealed that SAMD5 expression was associated with the cell cycle regulation of CC cell lines. *Conclusion*: SAMD5 is a novel marker for PBG but not LPC in mice. In humans, the expression and location of SAMD5 could become a promising diagnostic marker for the cell type as well as malignancy of bile ducts and CCs.

## Introduction

Bile ducts, which consist of biliary epithelial cells (BECs) or “cholangiocytes” functions to excrete bile from the hepatic parenchyma to the duodenum. While the intrahepatic bile ducts branch and connect with the bile canalicular system of hepatocytes in liver, the extrahepatic bile duct links the intrahepatic bile tract to the duodenum. It is known that the characteristics of BECs such as morphology, functions and gene expression patterns, are heterogeneous at the distinct anatomical location of the biliary tree [1, 2]. For example, cuboidal BECs constitute the peripheral small bile duct or ductules in liver, while columnar BECs lining large bile duct at the hepatic hilum or extrahepatic bile duct produce mucin. In addition, the peripheral branches of intrahepatic bile duct expand in chronically injured liver, which is known as ductular reactions [3]. Adult liver stem/progenitor cells (LPCs) are assumed to reside in a compartment of such biliary ductules [4,5]. By contrast, the extrahepatic bile duct including common bile duct bears accessory glands, called “peribiliary glands (PBGs)” [6,7]. PBGs have been implicated in the development and diseases of the hepatobiliary and pancreatic systems [8–10]. Recently, it has been reported that PBGs contain multi-potent stem/progenitor cells, called “biliary tree stem/progenitor cells (BTSCs)”, which give rise to hepatocytes, BECs and pancreatic islets [11]. Although much effort has been made to uncover the nature of LPCs and BTSCs respectively, the characteristic difference between intra- and extra-hepatic bile ducts in pathological process needs further investigation.

Biliary diseases such as primary sclerosing cholangitis and primary biliary cirrhosis are usually accompanied by severe cholestasis. Among mouse models, long-term feeding of 3,5-diethoxycarbonyl-1,4-dihydro-collidine (DDC) is one of the conventional methods to study cholestatic liver disease [12], and DDC-fed mice exhibit ductular reaction as well as chronic cholestasis [13,14]. We previously reported that Epithelial cell adhesion molecule (EpCAM) is expressed in mouse hepatoblasts, i.e. fetal LPCs as well as BECs during liver development [15], and that EpCAM+ cells sorted from normal and injured adult livers contain bi-potent liver stem cell-like cells which differentiate into both hepatocytic and biliary cells *in vitro* [16]. Further microarray analyses comparing gene expression profiles of EpCAM^+^ cells between normal and DDC-fed mouse livers have led to two findings that Nephronectin exacerbates liver injury in acute and chronic hepatitis [17] and that Semaphorin 3E regulates sinusoidal regeneration and liver fibrosis [18]. Although Sterile alpha motif domain containing 5 (SAMD5) was identified as one of such upregulated genes in EpCAM^+^ cells of DDC-fed mouse liver, the role of SAMD5 in liver diseases remained uninvestigated.

SAMD5 is one of the SAM domain-containing proteins. The SAM domain spreads over around 70 residues and has diverse roles for cellular processes via polymerization [19–21]. Different SAM domains can self-associate [22], and bind to other SAM domains [23] as well as other non-SAM proteins [24], RNA, DNA [25,26] or even lipids [27]. Although the functions of SAMD5 are entirely unknown, previous study demonstrated that pituitary homeobox 2 (*PITX2*) knockdown downregulates SAMD5 expression in primary human trabecular meshwork cells [28]. Watanabe *et al*. revealed that SAMD5 expression is closely associated with therapeutic efficiency of chemo-radiotherapy for rectal cancer [29]. These studies suggest that SAMD5 expression is relevant to the pathological staging or diagnosis. However, the expression profile and role of SAMD5 in biliary diseases including cholangiocarcinoma (CC) remain totally unknown.

We herein demonstrate that SAMD5 is drastically upregulated in large bile tracts including perihilar and exptrahepatic bile ducts in DDC-fed mouse livers whereas neither intrahepatic cuboidal BECs nor LPCs express SAMD5. More interestingly, SAMD5 is expressed in PBGs in normal mice without injury. By contrast, human SAMD5 is highly expressed and localized in the nucleus of CCs, while it is moderately observed in the cytoplasm of normal cholangiocytes lining hilar large bile ducts. Functional studies further demonstrate that SAMD5 expression is associated with cell cycle regulation of CC cell line *in vitro*. Our data suggest that SAMD5 is a useful marker for identifying the origin of biliary cells derived from BTSCs or LPCs and that its intracellular localization and expression level in CCs may be a diagnostic indicator for their proliferative and pathological situation.

## Materials and methods

### Mice and cell line

C57BL/6 mice were purchased from CLEA Japan Inc. (Tokyo, Japan) and used for all experiments. All animal experiments were performed in accordance with the guidelines approved by the Institutional Animal Care and Use Committee of the University of Tokyo. The CC and Hepatocellular carcinoma (HCC) cell lines were from RIKEN BRC. The RBE cell line was a kind gift from Dr. Munechika Enjoji.

### Liver injury and partial hepatectomy

Chronic liver injury was induced by feeding 0.1% DDC-containing diet (CLEA Japan Inc., Tokyo, Japan) or intraperitoneal injection of carbon tetrachloride (CCl_4_). CCl_4_ (Wako Pure Chemical, Osaka, Japan) was diluted in corn oil (Wako Pure Chemical, Tokyo, Japan) to 20% and injected into mice at a dose of 1-ml CCl_4_/kg body weight. Livers were harvested after feeding of DDC-diet for 2 weeks or repeated injections of CCl_4_, twice per week for 4 weeks, followed by euthanasia with cervical dislocation. 70% Partial hepatectomy (PHx) was performed as described previously [30]. Anesthesia was induced by sevoflurane inhalation. Following euthanasia, livers were harvested after 2 days from the operation. To examine expression level of Samd5 by real-time RT-PCR, liver samples of normal livers (n = 3), 70% PHx livers (n = 4), and chronically injured livers by CCl_4_ (n = 5) or DDC diet (n = 4) were analyzed. Normal livers of untreated 8 weeks old male mice were used as controls. We have observed no clinical symptoms nor signs of suffering during the experiment.

### RT-PCR and quantitative RT-PCR

Total RNA was isolated from mouse livers or hepatic cells using TRIzol reagent (Invitrogen, Carlsbad, CA). Reverse-transcription to cDNA templates was performed using random primers and a High-capacity cDNA Reverse-Transcription Kit (Applied Biosystems, Foster City, CA). Real-time RT-PCR experiments were conducted with a LightCycler 480 system and Universal Probe Library (Roche Diagnostics, Indianapolis, IN). The *ACTB* or *GAPDH* gene assay in Probe Library was used as the normalizing control. The sequence information for the primer pairs and probes used is listed in S1 Table.

### Isolation of EpCAM^+^ cells from livers and FACS analysis

EpCAM^+^ cells were isolated from murine livers as described previously [16]. Aliquots of non-parenchymal cells were blocked with anti-FcR antibody and incubated with biotin-conjugated anti-EpCAM monoclonal antibody on ice. Then, cell suspension was washed and incubated with allophycocyanin-conjugated streptavidin (BD Biosciences, San Diego, CA). EpCAM^+^ cells were roughly sorted by autoMACS pro (Miltenyi Biotec, Bergisch Gladbach, Germany) with anti-APC microbeads and purified by fluorescence-activated cell sorting (FACS) using Moflo XDP (Beckman-Coulter, Fullerton, CA). Dead cells were excluded by propidium iodide staining.

### Generation of anti-SAMD5 polyclonal antibody

Rabbit anti-SAMD5 polyclonal antibody was raised as previously described [31]. In brief, cDNA encoding mouse SAMD5 was cloned from total RNA of DDC-fed mice liver by RT-PCR using the following primers (sense, 5’-GGA TCC CGA GTC TCA GCC ATG TGC-3’, and antisense, 5’-GTC GAC CAA AAA TGA TAT CTA GTG G-3’). The cDNA was subcloned into pET-32a vector (Merck Millipore, Billerica, MA) or pGEX-4T-3 vector (GE Healthcare Life Sciences, Piscataway, NJ). The expression vectors for tagged fusion proteins, His-SAMD5 and GST-SAMD5 were over-expressed in the BL21 *Escherichia coli* strain. His-SAMD5 was affinity-purified by HisTrap HP (GE Healthcare Life Sciences) and used for immunization using rabbits. Anti-SAMD5 antibody was affinity-purified from the rabbit serum by using HiTrap NHS-activated HP columns (GE Healthcare Life Sciences) coupled with GST-SAMD5. The cross-reactivity of anti-SAMD5 antibody to mouse and human SAMD5 was confirmed by Western blot analysis using the cell lysate of Cos-7 transfected with mouse or human *SAMD5* cDNA expression vector (S1 Fig).

### Immunohistochemistry and Periodic Acid-Schiff (PAS) staining

Eight-micrometer liver cryosections were mounted on glass slides and fixed with Zamboni’s fixative solution for 10 min for immunohistochemistry (IHC) staining. The fixed sections were incubated with 5% skim milk (w/v) in PBS and then incubated with primary antibodies, followed by secondary antibodies. The antibodies used in this study are described in Table 1. Images were captured using Observer Z1 with an AxioCam HRc (Zeiss, Oberkochen, Germany). Periodic acid-Schiff (PAS) staining was performed for serial section of IHC-stained section. The fixed sections were exposed to orthoperiodic acid (Wako Pure Chemical, Tokyo, Japan) and then stained with Schiff’s Reagent (Muto Pure Chemicals, Tokyo, Japan). Sulfite Solution (Muto Pure Chemicals, Tokyo, Japan) was used for wash.

**Table 1:** Primary antibodies.

Antibodies	Company/Producer	Cat. Number	Dilution
ACTB	Santa Cruz	sc-1616	1:200
CK19	DSHB	TROMA-III	1:200
EpCAM	BD Pharmingen	552370	1:100
FLAG	Sigma	F3165	1:200

### Knockdown of SAMD5 and cell cycle analysis

Stealth RNAi siRNA for human SAMD5 was purchased from Life Technologies (Carlsbad, CA), and negative Universal Control was used as a control for all knockdown experiments. The siRNAs were introduced into the cell by Lipofectamine RNAiMAX Transfection Reagent (Life Technologies, Carlsbad, CA). Cell cycle analysis was performed as previously described [30].

### Forced expression of SAMD5

Human SAMD5 expression vector was constructed by using primer pairs listed in S1 Table as “Human SAMD5 for expression vector”. Amplified SAMD5-coding sequence was assembled to pCMV-FLAG vector. The vector was introduced into the cells by lipofection using polyethylenimine.

### Analysis of cell proliferation

WST-1 assay was performed for quantifying the status of cell proliferation. Cells were cultured in 96-well culture dish and WST-1 reagent (Dojindo, Kumamoto, Japan) was applied for the culture medium. After 4 hours of incubation, the difference between absorbance at 440 nm and 600 nm of the medium was measured.

### Liver tissue specimens and immunohistochemistry

Surgically resected liver specimens including cholangiocarcinoma (CC) in intrahepatic or extrahepatic biliary tree were used in this study. After fixation 10% neutral-buffered formalin, 4μm-thick sections were prepared. The deparaffinized and rehydrated sections were heat-treated in 10mM citrate buffer (pH 6.0) for 20 min at 95°C for the pretreatment of tissue prior to staining. Following endogenous peroxidase blocking and incubation in normal goat serum (dilute 1:10; Vector Lab, Burlingame, CA) for 20 minutes, these sections were incubated at 4°C overnight with rabbit polyclonal SAMD5 antibody (1μg/mL), and then at room temperature for 1 hour with goat anti-rabbit immunoglobulins conjugated to peroxidase labeled-dextran polymer (Envision+TM, Dako Japan). After benzidine reaction, sections were counterstained with hematoxylin. As a negative control, normal rabbit IgG (1μg/mL) was used for the primary antibody. This procedure consistently resulted in no staining.

### Preparation of normal human BEC samples

Normal human BECs were isolated, purified and cultured from human liver specimens, as described previously [32]. Human BECs were incubated with a culture medium composed of D-MEM/F-12, Nu-Serum (Becton Dickinson, Bedford, MA), ITS+ (Becton Dickinson), 5μM forskolin (Wako, Osaka, Japan), 12.5mg/ml of bovine pituitary extract (Gibco, Carlsbad, CA), 1μM dexamethasone (Sigma, St Louis, MO), 5μM Triiodo-thyronine (Sigma), 5mg/ml glucose (Sigma), 25mM sodium bicarbonate (Sigma), 1% antibiotics antimycotic, 20ng/ml of human epidermal growth factor (Gibco), and 10ng/ml human hepatocyte growth factor (Gibco). Total RNA prepared from the cultured BECs within 10 passages (n = 3) were used for quantitative RT-PCR analysis.

### Study approval

All mouse studies were conducted in accordance with institutional procedures and approved by the Animal Care and Use committee of the Institute of Molecular and Cellular Biosciences, The University of Tokyo (approval numbers 2501, 2501–1, 2609 and 2706) and for the National Center for Global Health and Medicine Research Institute (approval number 16029). The study using human samples was approved by the Kanazawa University Ethics Committee (approval number 305–4), and all of the analyzed samples are derived from patients who provided informed written consent for the use of their tissue samples in research.

### Statistical analysis

Statistical analysis was performed using the unpaired two-tailed Student’s *t*-test. A value of *P* <0.05 was taken to indicate statistical significance.

## Results

### EpCAM^+^ cells express SAMD5 in chronically injured mouse livers

**Fig 1:**
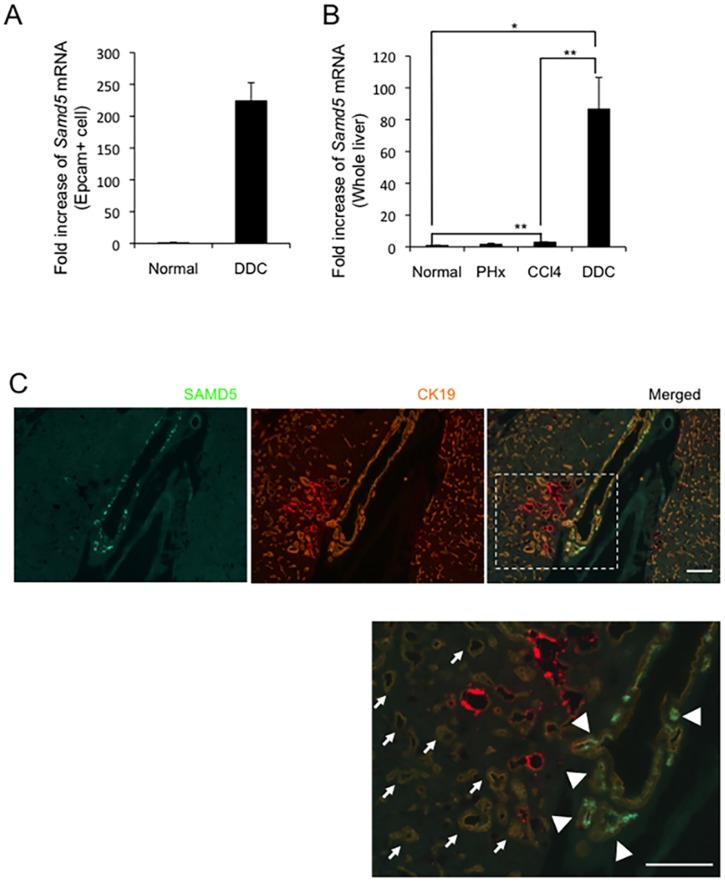
Expression profiles of SAMD5 in mouse. (A). Real-time RT-PCR analysis of Samd5 mRNA in EpCAM^+^ cells isolated from normal and DDC-fed mouse livers. (B) Real-time RT-PCR analysis of Samd5 mRNA in normal liver (n = 3), 70% PHx liver (n = 4), chronically injured liver by CCl_4_ (n = 5) or DDC diet (n = 4). The upregulation of SAMD5 expression is outstanding in DDC-fed mouse liver. Data are means ± standard error. *P <0.05; **P <0.01. (C). Immunostaining of SAMD5 and CK19 for DDC-fed mouse liver. SAMD5 is markedly expressed in a part of large bile ducts and PBGs at the hepatic hilum (arrowheads), whereas it is not detected in numerous ductular cells located in parenchymal region (arrows). Bars = 50 μm.

We have reported previously that potential LPCs reside in EpCAM^+^ cells in DDC-fed mouse livers [16]. The cDNA microarray analysis of EpCAM^+^ cells from normal and DDC-treated livers revealed that many genes including *Samd5* were upregulated in DDC-treated EpCAM^+^ cells compared to normal EpCAM^+^ cells (Table 2). Quantitative RT-PCR analysis demonstrated that SAMD5 expression of DDC-treated EpCAM^+^ cells is more than 200 times higher than that of normal EpCAM^+^ cells (Fig 1A). Furthermore, the expression levels of SAMD5 in various liver injury models were determined by quantitative RT-PCR. SAMD5 was slightly but significantly upregulated in chronically injured livers by repeated injections of carbon tetrachloride (CCl_4_), but not in regenerating livers after partial hepatectomy (PHx) (Fig 1B). By contrast, *Samd5* mRNA was markedly upregulated in DDC-fed livers, suggesting that its expression was induced in severely cholestatic livers.

**Table 2:** Result of cDNA microarray analysis of EpCAM^+^ cells between normal and DDC-fed mouse livers.

	Raw signal intensity of EpCAM^+^ cells		
Gene Name	Normal liver	DDC liver	DDC/Normal Ratio
chitinase 3-like 3	0.6	319.2	570.0
chitinase 3-like 4	0.7	284.0	399.2
cadherin 17	1.8	602.0	343.0
glucosaminyl (N-acetyl) transferase 3, mucin type	0.6	162.8	280.5
**sterile alpha motif domain containing 5**	1.9	448.5	241.0
plexin domain containing 2	1.2	247.5	210.4
serine (or cysteine) peptidase inhibitor, clade B, member 5	0.9	157.8	168.2
THO complex 1	0.2	22.2	139.9
centromere protein E	1.1	141.5	132.7
ATPase, Class V, type 10B	1.8	236.8	128.1
ST8 alpha-N-acetyl-neuraminide alpha-2,8-sialyltransferase 6	3.6	439.2	123.5
E2F transcription factor 8	1.0	126.3	123.4
runt related transcription factor 1	0.7	86.5	122.7
sema domain, immunoglobulin domain (Ig), short basic domain, secreted, (semaphorin) 3E	1.1	132.2	117.9
tumor-associated calcium signal transducer 2	2.4	284.3	117.2
asparagine synthetase	0.8	93.6	115.3
dihydropyrimidinase-like 3	1.3	135.8	104.3
regulator of G-protein signaling 4	0.7	74.2	103.8
trichohyalin	0.4	35.6	99.4
cyclin B1, related sequence 1 /// cyclin B1	4.0	394.2	99.2

### Expression of SAMD5 is observed in BECs consisting of dilated large bile duct and PBG

To investigate the location of SAMD5-expressing cells *in vivo*, we generated anti-SAMD5 polyclonal antibody for immunostaining by immunizing a rabbit with GST-fused mouse SAMD5 protein. Because the *Samd5* expression in kidneys was comparable to that of livers (S2A Fig), we first evaluated the anti-SAMD5 antibody by immunohistochemistry (IHC) analysis using frozen kidney sections. As shown in S2B Fig, renal glomeruli were clearly stained by the anti-SAMD5 antibody. Next, double immunostaining against SAMD5 and Cytokeratin 19 (CK19), another marker for BECs and LPCs, was performed for normal mouse livers. While intrahepatic bile ductules were not stained by the anti-SAMD5 antibody, faint signal of SAMD5 was detected in CK19+ cells adjacent to the large bile duct at the hepatic hilum, suggesting that SAMD5 is expressed in intramural accessory glands of perihilar large bile ducts (S2C and S2D Fig). By contrast, IHC analysis of DDC-fed livers revealed that SAMD5 was predominantly expressed in the intrahepatic large bile duct at the hepatic hilum, while proliferating atypical ductular cells including LPCs seemed to show no staining of SAMD5 (Fig 1C). Interestingly, intense signals of SAMD5 were detected in the acini of PBG of hilar large bile ducts. These results suggested that SAMD5 was upregulated in the epithelial cells of intrahepatic large bile ducts and PBG at the hepatic hilum after DDC-induced injury, but neither BECs lining intrahepatic small tubules nor proliferating LPCs expressed SAMD5.

### SAMD5 expression is correlated with mucus producing BECs

**Fig 2:**
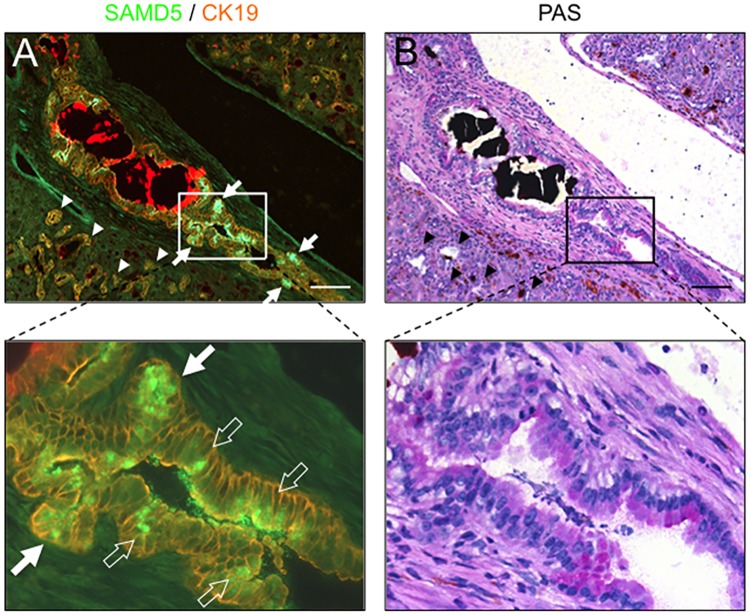
IHC and PAS staining for serial sections of DDC-fed mice liver. (A) Immunostaining of SAMD5 and CK19 for DDC-fed mice liver. SAMD5 is markedly expressed in several PBGs (solid arrows) and columnar mucus-producing cholangiocytes (open arrows) at the hepatic hilum, whereas SAMD5 is not detected in cuboidal ductular cells (arrow heads). (B) PAS staining for the serial section of panel (A). Mucin is stained violet, while deposition of Iron and bile plug is observed as red and black agglutination. Mucin is detected in hilar large bile duct and PBG, but not in cuboidal ductular cells (arrow heads). The lower panel is a magnified image of the upper panel. Bars = 100 μm.

Because the previous report demonstrated that columnar BECs consisting the intrahepatic large bile duct, hilar bile duct and extrahepatic bile duct produce mucus in human livers [33]. we examined the correlation between SAMD5 expression and mucus production in DDC-fed mice. Serial sections of DDC-fed mouse livers were subjected to IHC staining using anti-SAMD5 antibody and Periodic Acid-Schiff (PAS) staining, a method to detect polysaccharides including mucus, respectively (Fig 2). It was revealed that several hilar columnar BECs and acini producing mucus in DDC-fed livers expressed SAMD5 at the apical region of cytoplasm, while mucin-negative cuboidal LPCs did not. These results suggested that SAMD5 expression might be associated with mucus production or define the cell lineage of columnar cholangiocytes in DDC-fed cholestatic livers.

### SAMD5 is exclusively expressed in PBG of normal extrahepatic bile ducts

**Fig 3:**
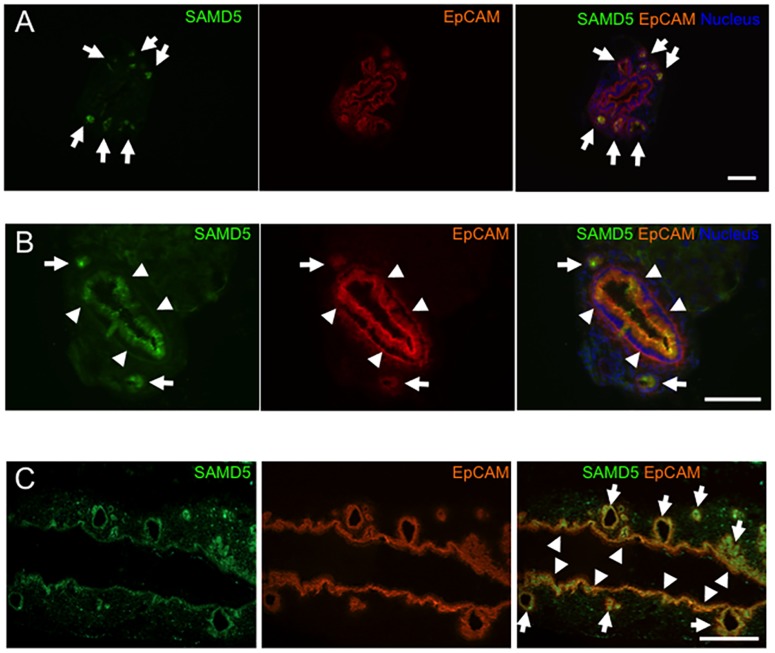
Expression profiles of SAMD5 and EpCAM in extrahepatic bile duct. (A) Double immunostaining of the vertical section of normal common bile duct with anti-SAMD5 and anti-EpCAM antibodies. SAMD5 is clearly detected in PBGs (arrows), but not in the luminal epithelium of normal common bile duct. (B, C) Double immunostaining of the vertical (B) and horizontal (C) sections of DDC-fed common bile duct with anti-SAMD5 and anti-EpCAM antibodies. SAMD5 is highly expressed in both PBGs (arrows) and the luminal epithelium of dilated common bile duct (arrowheads).

Because PBGs are located around the extrahepatic bile duct as well as the hilar large bile duct, we investigated the expression profile of SAMD5 in extrahepatic bile ducts. Intriguingly, IHC analysis of the vertical section of common bile ducts revealed that SAMD5 was exclusively expressed in PBGs of normal extrahepatic bile ducts (Fig 3A). To investigate whether SAMD5 is induced in extrahepatic cholangiocytes like perihilar large bile ducts by cholestatic injury, we performed IHC analysis of extrahepatic bile ducts after DDC treatment. Double staining of vertical and horizontal sections of common bile ducts with anti-SAMD5 and anti-EpCAM antibodies revealed that SAMD5 was highly expressed in not only PBG, but also in the apical region of the columnar cholangiocytes lining dilated common bile ducts (Fig 3B and 3C). These results suggested that SAMD5 is expressed in PBGs under normal condition, but induced in columnar cholangiocytes lining large bile ducts after cholestatic injury.

### SAMD5 is expressed in human columnar BECs and CCs

**Fig 4:**
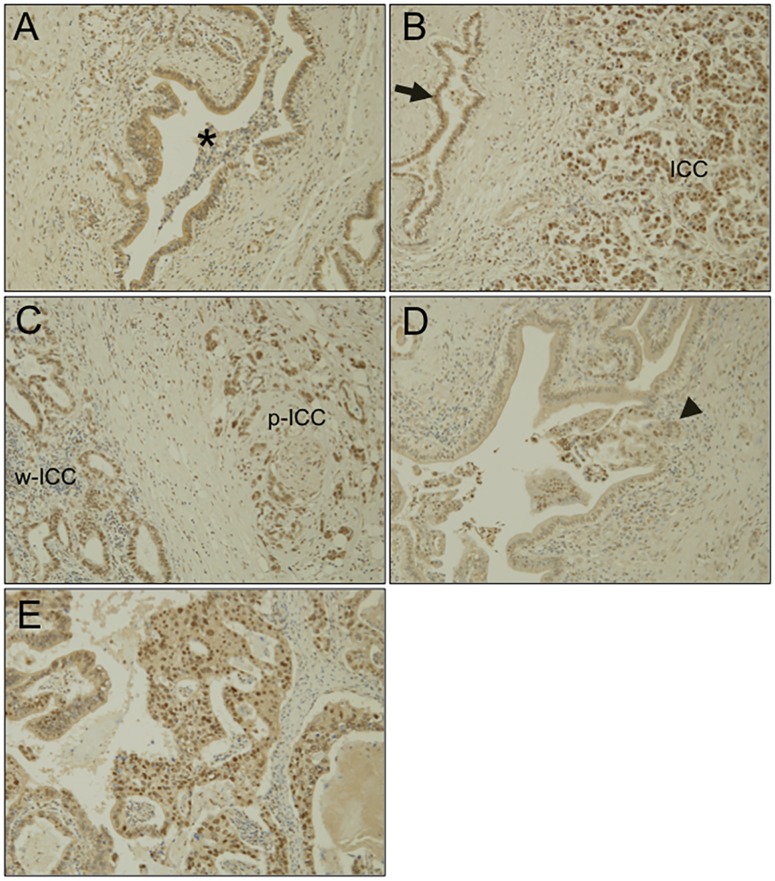
Immunostaining of human liver tissue sections with anti-SAMD5 antibody. SAMD5 was stained for the paraffin-embedded sections of normal large bile duct at the hepatic hilum (A), intrahepatic CC (B), hilar CC (C and D), and extrahepatic CC (E). (A) Low or medial cytoplasmic staining of SAMD5 is observed in normal hilar large bile duct (asterisk). (Original magnification X200) (B) While the intrahepatic cholangiocytes show the cytoplasic staining of SAMD5 (arrow), the poorly-differentiated ICC exhibits striking nuclear staining of SAMD5. (Original magnification X100) (C) SAMD5 is stained in the nuclear of both poorly-differentiated ICC (p-ICC) and well-differentiated ICC (w-ICC) at the hepatic hilum. (Original magnification X200) (D) The cancerous cells invading hilar large bile duct show nuclear staining of SAMD5 (arrowhead). (Original magnification X200) (E) The papillary and moderately-differentiated tubular adenocarcinomas in the common bile duct exhibit nuclear staining of SAMD5. (Original magnification X200).

In humans, it has been suggested that intrahepatic CC (ICC) could arise from intrahepatic PBG [34]. In addition, a recent work reported that mucin-producing ICCs had a clinicopathological, immunohistochemical, and molecular profile similar to that of hilar CCs (from mucin-producing cholangiocytes) [33]. Therefore, we next investigated the expression of SAMD5 in human CC specimens by immunostaining. Intriguingly, SAMD5 was observed at a slight-to-moderate level in the cytoplasm of normal columnar BECs lining large bile ducts at the hepatic hilum (Fig 4A). By contrast, surprisingly, the nuclear staining of SAMD5 in ICCs was observed in five out of six examined hilar CC specimens. SAMD5 was localized in the nucleus of poorly-differentiated ICCs (Fig 4B). In addition, the nuclear localization of SAMD5 was also observed in both well-differentiated and poorly-differentiated ICCs at the hepatic hilum (Fig 4C). Interestingly, the cancerous cells invading the hilar large bile duct exhibited apparent nuclear staining of SAMD5 (Fig 4D). In addition, the papillary and moderately-differentiated tubular adenocarcinoma in the common bile duct of extrahepatic CC also showed nuclear staining of SAMD5 (Fig 4E). These results suggested that the localization of SAMD5 is quite distinct between normal BECs and CCs, which may be involved in the promotion of carcinogenesis.

### Expression of SAMD5 in CC cell lines

**Fig 5:**
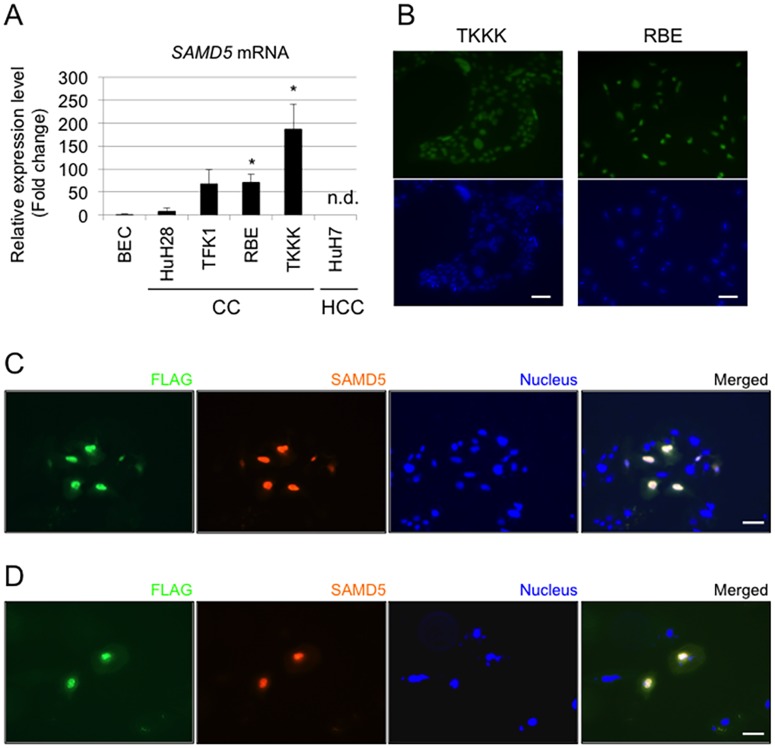
Expression profiles of SAMD5 in human HCC and CC cell lines. (A) the relative expression of *SAMD5* gene in four CC cell lines (HuH28, TFK1, RBE and TKKK) and one HCC cell line (HuH7) to normal biliary epithelial cell (BEC) by quantitative RT-PCR. *SAMD5* mRNA was increased in all CC cell lines, but not expressed in HuH7. (n = 3; *P <0.05, compared to BEC) Data are mean ± standard error. n.d.: not detected (B). Immunocytochemical images of SAMD5 for CC cell lines. SAMD5 is visualized and localized at the nuclei of TKKK and RBE. Bars = 50 μm. (C and D) Images of exogenously introduced FLAG-tagged SAMD5 in HuH7 (C) and HuH28 (D) by Immunocytochemical staining. Overexpressed SAMD5 translocated to the nuclei of each cell. Bars = 50 μm.

To further investigate the role of SAMD5 in CC cells, the expression level of *SAMD5* gene in four CC cell lines (HuH28, TFK1, RBE and TKKK) and one HCC cell line (HuH7) relative to normal BEC was examined. Quantitative RT-PCR revealed that *SAMD5* mRNA was increased in all CC cell lines compared to BEC, but not expressed in HuH7 (Fig 5A). The IHC staining of SAMD5 demonstrated that intense signals of SAMD5 were detected in the nuclei of TKKK, RBE and TFK1 (Fig 5B and S3 Fig). These results indicated that SAMD5 is localized in the nuclei of CC cell lines as well as CC specimens.

### Exogenously expressed SAMD5 localizes to the nucleus of CC and HCC cell lines

Given that the localization of SAMD5 in the nucleus is relevant to some characteristics of carcinoma, exogenously expressed SAMD5 in HCC and/or CC cell lines could translocate to the nucleus. Therefore, we constructed the expression vector for human SAMD5 with FLAG-tag, and transduced it to HuH7 and HuH28 by lipofection, respectively. After 48 hours from lipofection, cell extracts were subjected to Western blot analysis by anti-SAMD5 antibody to confirm that SAMD5 protein was efficiently expressed (S4 Fig). Immunocytochemical staining of FLAG-tag and SAMD5 revealed that FLAG-tagged SAMD5 was translocated to the nucleus in both HuH7 and HuH28 (Fig 5C and 5D). These data suggested that SAMD5 was actively transported to the nucleus by itself or binding to other nuclear proteins in cancer cells.

### SAMD5 expression in cell cycle regulation and proliferation of CC

**Fig 6:**
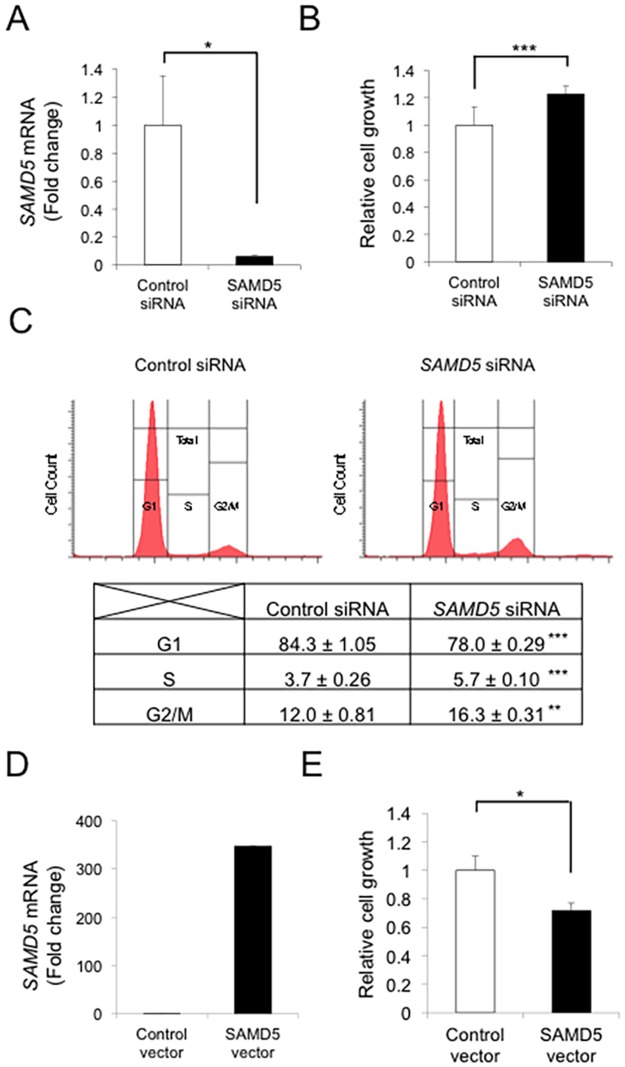
Relationship between SAMD5 expression and cell cycle in CC cell line. (A) Real-time RT-PCR analysis of *SAMD5* mRNA in RBE cell line after 96 hours of knockdown using siRNA. n = 4 per each group. (B) Examination of RBE cell proliferation by WST-1 assay. n = 8 per each group. (C) Cell cycle analysis of RBE cell line by FACS. The knockdown of *SAMD5* in RBE cell line resulted in significant increase of cell population at S and M/G2 phase compared to the control. n = 3 per each group. (D) Real-time RT-PCR analysis of *SAMD5* mRNA in HuH28 cell line after 96 hours of overexpression. (E) Examination of HuH28 cell proliferation by WST-1 assay. n = 8 per each group. Data are mean ± standard error. *P <0.05; **P <0.01; ***P <0.001.

It has been reported previously that the SAM domain of SAMD4B regulates transcriptional activity of cell cycle-related genes such as AP-1, p53 and p21 [35]. Considering that SAMD5 was also localized in the nucleus of CCs, we hypothesized that SAMD5 might be involved in the cell cycle regulation of CCs. To address the hypothesis, we knocked down *SAMD5* mRNA in RBE cell line using siRNAs. We tested three distinct siRNA sequences for SAMD5 and evaluated their knockdown efficiency by quantitative RT-PCR analysis 48 hours after lipofection. Among them, siRNA #1 worked most efficiently (S5A Fig) and decreased *SAMD5* mRNA by 94% even 96 hours after lipofection (Fig 6A). To investigate the role of SAMD5 in the growth of RBE cell line, we performed WST-1 and FACS analysis 96 hours after knockdown of *SAMD5*. The WST-1 assay revealed that knockdown of *SAMD5* accelerated the proliferation of RBE (Fig 6B and S5B Fig). Consistently, FACS analysis demonstrated that knockdown of *SAMD5* significantly increased the cell population at S and G2/M phase compared to control siRNA (Fig 6C).

We next investigated the effect of SAMD5 overexpression on the growth of CC cell line. Because HuH28 exhibited relatively low expression of SAMD5 among four examined CC lines (Fig 5A), we introduced the expression vector for SAMD5 in HuH28 cell and evaluated cell growth after 96 hours of overexpression. WST-1 assay revealed that SAMD5 overexpression suppressed cell growth of HuH28 significantly compared to control (empty vector) (Fig 6D and 6E). These results suggested that SAMD5 played a role in the cell cycle regulation of CCs.

## Discussion

CC is a type of relatively rare neoplasm in adenocarcinoma and the etiology remains poorly understood. CC is an incurable malignancy unless the primary tumor can be fully resected. The lack of early prognostic marker for CC makes it difficult to diagnose CC at an early stage. In addition, ICCs are composed of heterogeneous carcinomas arising from different sites of biliary tree. Although ICCs have been basically subdivided into two types: the perihilar large duct type and the peripheral small duct type [36], further clinicopathological study using specific markers is required to define the cell type of origin. Because BTSC and LPC are assumed to exist at the PBGs of large bile ducts and at the canals of Hering of peripheral small bile ducts, ICCs could be derived from these cells [37,38]. It has been suggested that LPC is involved in the pathogenesis of cholangiocellular carcinoma with mixed features, while BTSC could be a origin of mucin-producing CCs.

In this study, we demonstrated that SAMD5 was expressed in PBGs of normal mice and upregulated in both mucin-producing PBGs and BECs lining intrahepatic large bile duct at the hepatic hilum, extrahepatic bile ducts of cholestatic mice. SAMD5 was identified by microarray analysis as an upregulated gene in EpCAM+ epithelial cells of DDC-fed mouse livers compared to those of normal livers. Because these EpCAM+ cells include cholangiocytes and LPCs, SAMD5 was initially expected to be a marker for LPC. However, neither intrahepatic cuboidal cholangiocytes nor LPCs expressed SAMD5 irrespective of liver injury. Because we removed extrahepatic bile ducts from livers to prepare EpCAM+ cells for microarray analysis, it is likely that the drastic upregulation of SAMD5 in sorted EpCAM+ cells is caused by the contamination of hilar columnar cholangiocytes lining large bile ducts and intramural PGBs of cholestatic livers (Fig 1C). These results suggested that SAMD5 expression might be a characteristic of the epithelial cell lineage constituting large bile ducts including perihilar and extrahepatic bile ducts, but not peripheral intrahepatic bile ducts. More interestingly, we found that mouse SAMD5 expression was restricted in PBG of intra- and extrahepatic large bile ducts in the absence of hepatic injury, and associated with mucus production. Considering that BTSCs are associated with mucin-producing cells [39], our findings suggested that SAMD5 could be a novel marker for mouse BTSC located in the PGBs. Recent emerging evidence from many pathological reports have suggested that the epithelial cells residing in PBG is involved in the stem cell compartment of biliary tree [11] or cholangiocellular carcinogenesis in human [9,10,34]. Considering that neither peripheral cuboidal BECs nor LPCs expressed SAMD5, our findings supported the notion that mucin-producing CC arise from BTSCs in the PBGs, but not from peripheral intrahepatic bile ducts or LPCs. In addition, we assumed that human ICCs derived from PBG may express SAMD5. Actually, four examined CC cell lines, but not HCC line showed increased expression of *SAMD5* mRNA compared to normal BEC. Furthermore, striking nuclear staining of SAMD5 in ICC was demonstrated in five out of six human hilar CC specimens and one extrahepatic CC while indistinct cytoplasmic staining was observed in normal perihilar bile duct. These results strongly suggested that not only the upregulation of *SAMD5* gene but also its translocation from cytoplasm to nucleus might be implicated in the malignancy of bile ducts. The potential nuclear localization signals (NLS) estimated by cNLS Mapper [40] locate at the C terminus of SAMD5. We demonstrated that exogenously overexpressed SAMD5 localized to the nucleus of CC and HCC cell lines. Because the NLS score is not very high (maximum score is 4.3), SAMD5 may be carried into the nucleus through binding to the other nuclear localization factors via SAM domain.

Although the functional role of SAMD5 remains largely unknown, Sa et al. reported very recently that an in frame fusion of *SAMD5* with *SASH1* was identified in 4 individual cases of skull base chordoma, implying the significance of SAMD5 in tumorigenesis [41]. On the other hand, Matsuo et al. have reported that the knockdown of *SAMD5* in small cell lung cancer (SCLC) cell lines by siRNA suppressed cell proliferation [42]. In contrast to the previous paper, we showed that knockdown and overexpression of *SAMD5* in CC cell lines resulted in enhancement and suppression of cell growth, respectively. Therefore, SAMD5 may have different functions depending on the context of cell type or its cellular location, although the localization of *SAMD5* in SCLC is unclear. Considering the cytosolic staining of SAMD5 in normal BECs, SAMD5 may originally serve as a tumor suppressor in the cytoplasm. If cytosolic SAMD5 plays a role in cell cycle arrest, it is possible that knockdown of SAMD5 will enhance cell growth, and that cytosolic SAMD5 overflowed from nuclear translocation due to overexpression will suppress cell growth. However, precise mechanisms underlying the cell cycle regulation of CC by SAMD5 need further investigation. The detailed analysis of SAMD5 localization in other tumor cells or overexpression of SAMD5 mutant lacking in nuclear import ability will be useful in dissecting the molecular mechanisms.

Taken together, this is the first report concerning the expression profile and function of SAMD5 in mouse PGB and human CC cells. Our data suggest that the expression and location of SAMD5 could be a potential diagnostic marker for identifying cell types as well as malignancy of bile ducts and CCs under pathological condition. Further investigation of SAMD5 function may lead to the development of prognostic prediction method of CC.

## Supporting information

S1 Fig Western blot analysis of cell lysates from Cos-7 expressing recombinant mouse or human SAMD5. The generated anti-SAMD5 antibody worked well in both SAMD5.
(TIF)

S2 Fig Expression profile of SAMD5 in mouse. (A) Expression analysis of Samd5 mRNA in various tissues by real-time RT-PCR. (B) IHC of frozen kidney section by anti-SAMD5 antibody. Renal glomeruli are clearly stained. (C, D) IHC of frozen liver section by anti-SAMD5 and anti-CK19. SAMD5 is not expressed in interlobular small bile ducts (C) and perihilar large bile duct (D), but in intramural PGB at the hepatic hilum (arrow). Scale bar: 100mm.
(TIF)

S3 Fig Expression profiles of SAMD5 in human CC cell lines. Immunocytochemical images of SAMD5 for CC cell lines. SAMD5 is visualized and localized at the nuclei of TFK1 and HuH28. Bars = 50 μm.
(TIF)

S4 Fig Western blot analysis of cell lysates from Huh7 and Huh28 expressing recombinant human SAMD5 by anti-SAMD5 antibody. (TIF)

S5 Fig Real-time RT-PCR analysis and WST-1 assay of RBE cell after knockdown of *SAMD5* mRNA. Three distinct sequences for *SAMD5* siRNA were adopted for knockdown experiment. (A) *SAMD5* siRNA #1 displayed the highest efficacy of knockdown 48 hours after lipofection by real-time RT-PCR. n = 3 per each group. (B) Knockdown of *SAMD5* in RBE cell showed the enhancement of cell growth by WST-1 assay after 96 hours of culture. n = 8 per each group. Data are mean ± standard error. *P <0.05; ***P <0.001.
(TIF)

S1 Table Primers and probes used for this study. (DOCX)
