# Supplementary figures and images for: Expression and localization of sterile alpha motif domain containing 5 is associated with cell type and malignancy of biliary tree
Source: PLoS One. 2017 Apr 7;12(4):e0175355. doi: 10.1371/journal.pone.0175355 (PMC5384680; doi:10.1371/journal.pone.0175355)

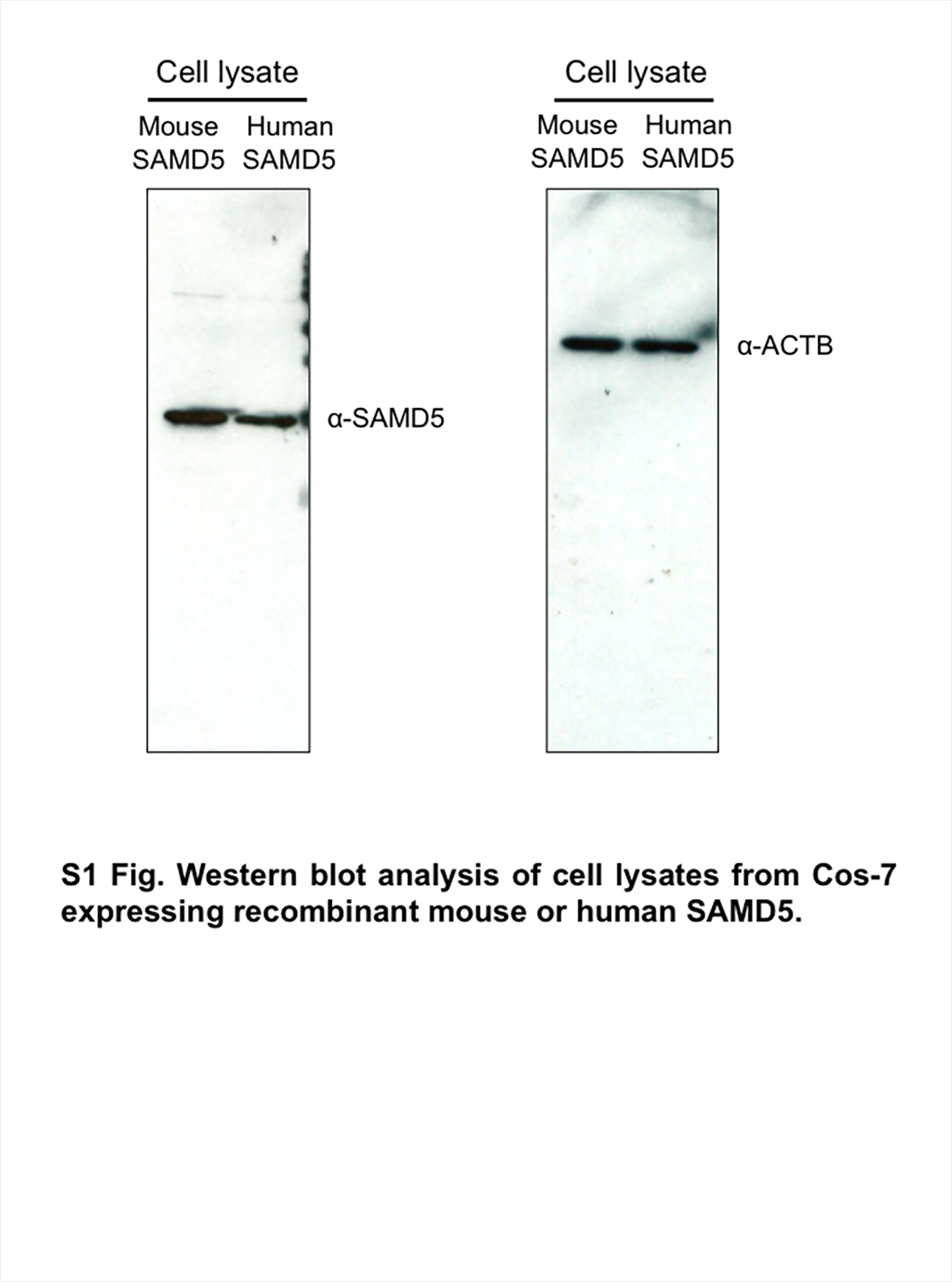

Supplement: S1 Fig — The generated anti-SAMD5 antibody worked well in both SAMD5. (TIF) [file pone.0175355.s001.tif]

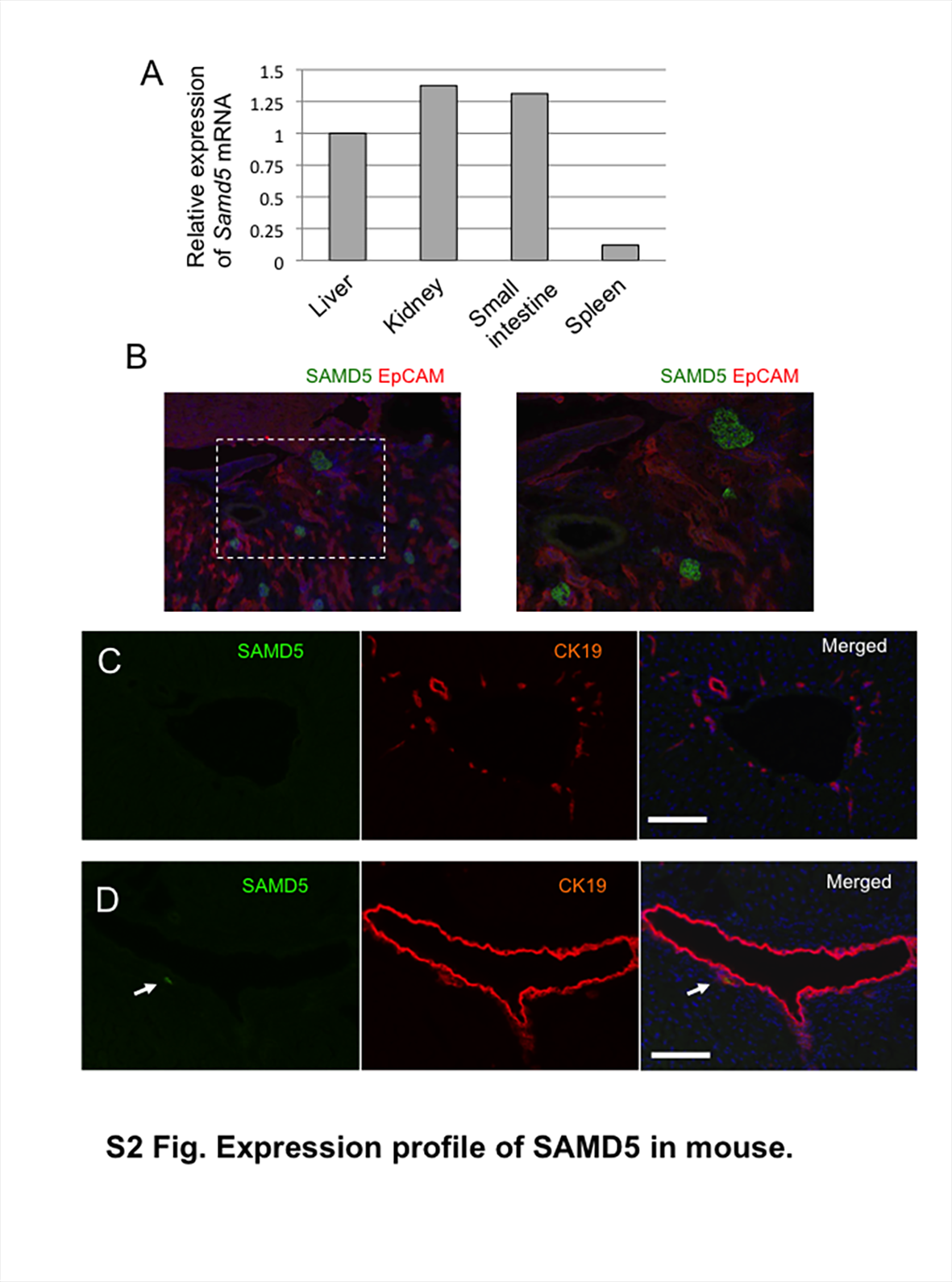

Supplement: S2 Fig — (A) Expression analysis of Samd5 mRNA in various tissues by real-time RT-PCR. (B) IHC of frozen kidney section by anti-SAMD5 antibody. Renal glomeruli are clearly stained. (C, D) IHC of frozen liver section by anti-SAMD5 and anti-CK19. SAMD5 is not expressed in interlobular small bile ducts (C) and perihilar large bile duct (D), but in intramural PGB at the hepatic hilum (arrow). Scale bar: 100mm. (TIF) [file pone.0175355.s002.tif]

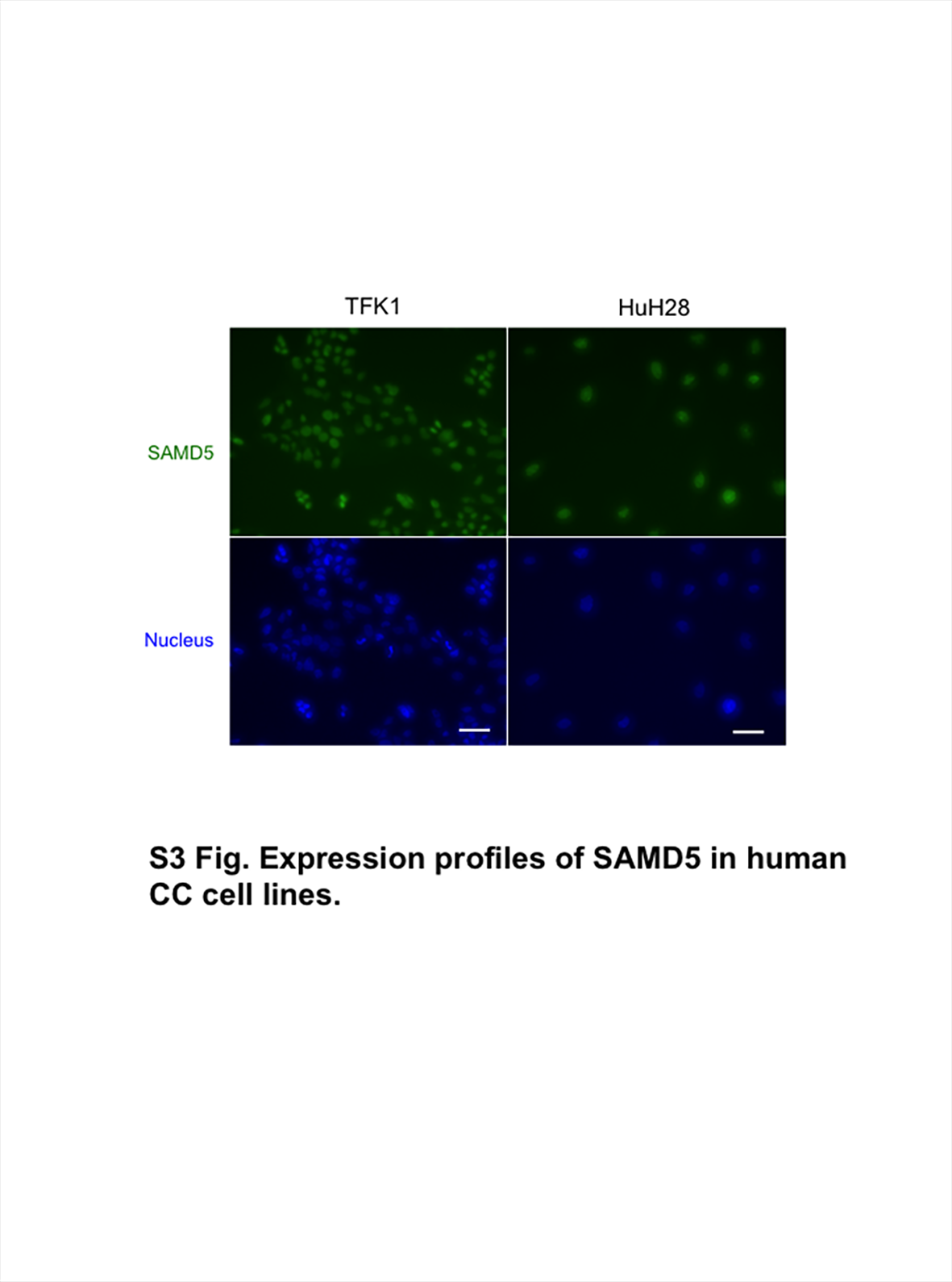

Supplement: S3 Fig — Immunocytochemical images of SAMD5 for CC cell lines. SAMD5 is visualized and localized at the nuclei of TFK1 and HuH28. Bars = 50 μm. (TIF) [file pone.0175355.s003.tif]

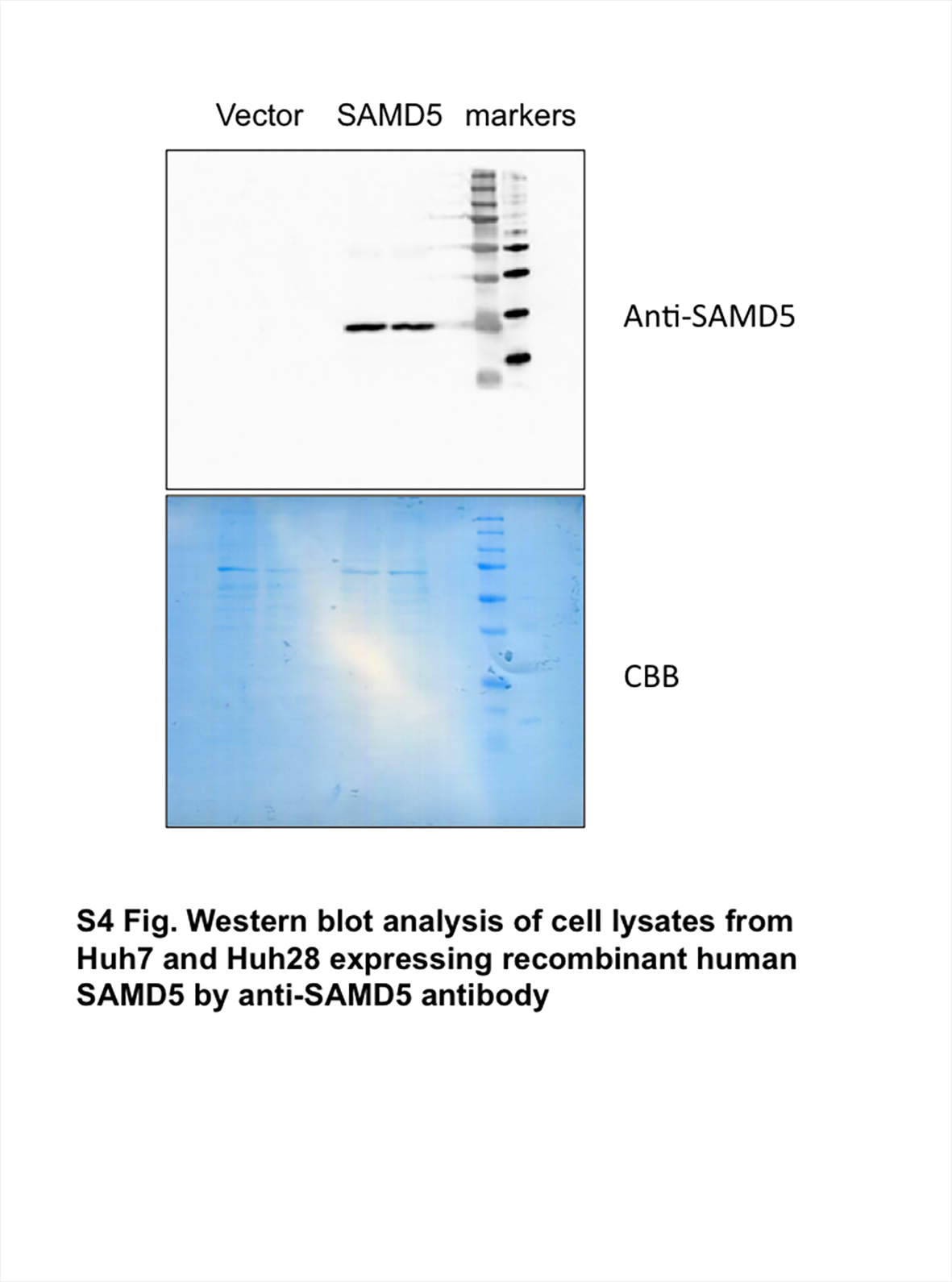

Supplement: S4 Fig — (TIF) [file pone.0175355.s004.tif]

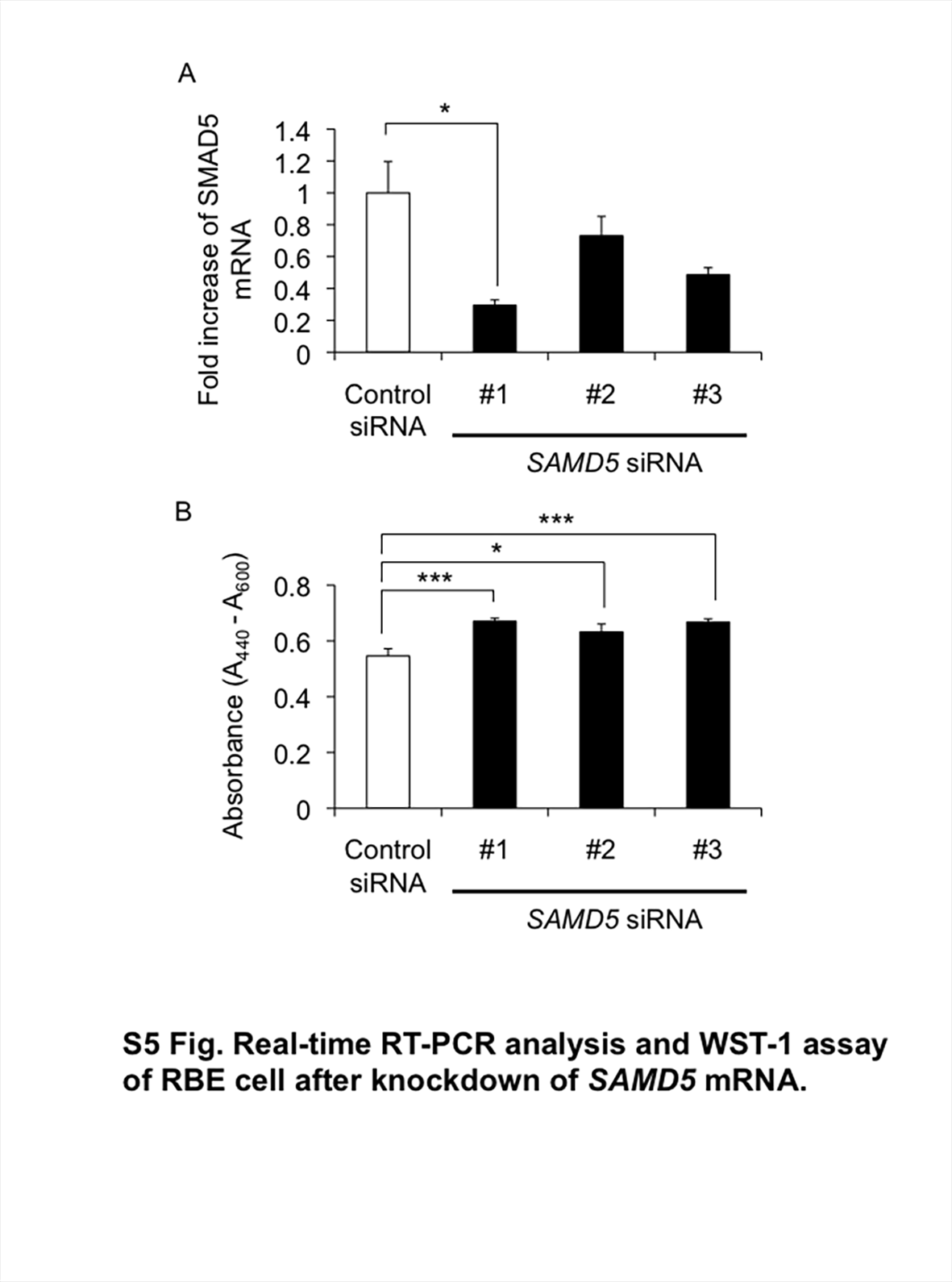

Supplement: S5 Fig — Three distinct sequences for SAMD5 siRNA were adopted for knockdown experiment. (A) SAMD5 siRNA #1 displayed the highest efficacy of knockdown 48 hours after lipofection by real-time RT-PCR. n = 3 per each group. (B) Knockdown of SAMD5 in RBE cell showed the enhancement of cell growth by WST-1 assay after 96 hours of culture. n = 8 per each group. Data are mean ± standard error. *P <0.05; ***P <0.001. (TIF) [file pone.0175355.s005.tif]
