# Supplementary material for: Expression and localization of sterile alpha motif domain containing 5 is associated with cell type and malignancy of biliary tree
Source: PLoS One. 2017 Apr 7;12(4):e0175355. doi: 10.1371/journal.pone.0175355 (PMC5384680; doi:10.1371/journal.pone.0175355)
Supplement: S1 Table — (DOCX) [file pone.0175355.s006.docx]

**S1 Table. Primers and Probes used for this study.**

| Gene | Sense primer sequence (5' to 3') | Antisense primer sequence (5' to 3') | Probe |
| --- | --- | --- | --- |
| Mouse SAMD5 | TTGGTTTTCAATGGACACTCC | GAGCAAAGGAGAGATTCCAA | GCAGGAAG |
| Human SAMD5 | GATCAGGGATAAGCTCGTCC | TCCACGAGGTCTCAAAAATG | GCTGGCATCCTAGAGTACTT |
| Human SAMD5 for expression vector | AGGATCCATGTGCACCAACATAGTTTAC | ACTCGAGTCAGTCCTCCACGAGGTCTC |  |
